# Correlates of isoniazid preventive therapy failure in child household contacts with infectious tuberculosis in high burden settings in Nairobi, Kenya – a cohort study

**DOI:** 10.1186/s12879-017-2719-8

**Published:** 2017-09-16

**Authors:** Florence Nafula Okwara, John Paul Oyore, Fred Nabwire Were, Samson Gwer

**Affiliations:** 1 Department of Pediatrics and Child Health, School of Medicine, Kenyatta University, 1609, Thika road campus, Nairobi, 0232 Kenya; 2 Department of Community Health, Kenyatta University, School of Public Health, Nairobi, Kenya; 3 Department of Paediatrics and Child Health, University of Nairobi, School of Medicine, Nairobi, Kenya; 4 Department of Medical physiology, Kenyatta University, Sechool of Medicin, Nairobi, Kenya; 5 Research and Evidence Program, Afya Research Africa, Nairobi, Kenya

**Keywords:** Tuberculosis, Children, Isoniazid, Prevention, Failure

## Abstract

**Background:**

Sub-Saharan Africa continues to document high pediatric tuberculosis (TB) burden, especially among the urban poor. One recommended preventive strategy involves tracking and isoniazid preventive therapy (IPT) for children under 5 years in close contact with infectious TB. However, sub-optimal effectiveness has been documented in diverse settings. We conducted a study to elucidate correlates to IPT strategy failure in children below 5 years in high burden settings.

**Methods:**

A prospective longitudinal cohort study was done in informal settlings in Nairobi, where children under 5 years in household contact with recently diagnosed smear positive TB adults were enrolled. Consent was sought. Structured questionnaires administered sought information on index case treatment, socio-demographics and TB knowledge. Contacts underwent baseline clinical screening exclude TB and/or pre-existing chronic conditions. Contacts were then put on daily isoniazid for 6 months and monitored for new TB disease, compliance and side effects. Follow-up continued for another 6 months.

**Results:**

At baseline, 428 contacts were screened, and 14(3.2%) had evidence of TB disease, hence excluded. Of 414 contacts put on IPT, 368 (88.8%) completed the 1 year follow-up. Operational challenges were reported by 258(70%) households, while 82(22%) reported side effects. Good compliance was documented in 89% (CI:80.2–96.2). By endpoint, 6(1.6%) contacts developed evidence of new TB disease and required definitive anti-tuberculosis therapy. The main factor associated with IPT failure was under-nutrition of contacts (*p* = 0.023).

**Conclusion:**

Under-nutrition was associated with IPT failure for child contacts below 5 years in high burden, resource limited settings. IPT effectiveness could be optimized through nutrition support of contacts.

**Electronic supplementary material:**

The online version of this article (10.1186/s12879-017-2719-8) contains supplementary material, which is available to authorized users.

## Background

Sub-Saharan Africa continues to document high burden of pediatric tuberculosis (TB), driven by the HIV epidemic [1]. The urban poor, especially those living in informal settlements have disproportionately higher risk, largely attributed to poverty, poor housing, migration and malnutrition [2]. Childhood TB constitutes up to 20–40% of case loads in certain communities [2]. Most (60%) childhood infections occur at household level [3]. Infected children experience rapid disease progression and severe forms of disease, as a result of their immature immune systems [4]. Various preventive strategies have been adopted in endemic settings. Widespread BCG vaccination has not slowed transmission in these high burden areas [5]. Household Contacts’ (HHC) tracing offers the best opportunity to identify children at risk and provide access to preventive therapy. World Health Organization (WHO) recommends 6 months isoniazid preventive therapy (IPT) for children under 5 years in close contact with infectious TB [6]. The success of IPT is dependent on complex interaction of numerous mediators including, magnitude of exposures, effective contact investigations, inherent contact vulnerabilities and mycobacterial infectivity [7]. High prevalence of Human immune deficiency (HIV) and TB co-morbidity among adults in informal settlements often results in continuous TB exposures. In a study done in South Africa, 10% of HIV exposed, uninfected children, had contact with infectious TB case by the age of 3–4 months [8]. There is evidence of effectiveness of IPT among HHC in studies from low burden countries [9, 10]. However, two meta-analysis observed marked heterogeneity in efficacy, with benefit observed among HIV negative, but not in the HIV-infected children [11, 12]. A South African study found no benefit of primary IPT in the vulnerable young population of HIV exposed children [13]. In Uganda, 1% IPT failure was observed in children exposed to smear positive index cases [14]. There is paucity of information on factors contributing to these inconsistencies among young children in high burden settings. Prevailing operational drawbacks may play a major role [13]. We conducted a study to elucidate factors associated with IPT strategy failure in child HHC with smear positive adults from informal settlements within Nairobi, Kenya.

## Methods

### Study design and settings

We conducted a multi-center prospective longitudinal cohort study at three public health facilities located within informal settlements in Nairobi (Kayole, Dandora and Mukuru), Kenya. These sites recorded among the highest new TB case detection rates in Nairobi, according to the Kenya Division of Leprosy, TB and lung disease (DLTLD) program report of 2010 (unpublished data). The study centers offered primary health care services and were registered TB diagnostic and treatment centers by the DLTLD. Recommendations for contacts’ tracking and IPT for under five HHCs was entrenched into the Kenya pediatric TB guidelines in 2010. By start of this study, none of these sites had implemented the strategy. The Mbagathi District Hospital: a secondary level referral heath facility, was the approved reference laboratory for the sites. Subject enrollment started from December 2011 to December 2012, but follow up continued until July 2013. Recently diagnosed (within 1 month) smear positive TB index cases were identified from TB clinic registers, and were invited to bring their contacts to a contacts’ clinic held weekly.

### Study population

We enrolled children aged below 5 years, living and sleeping under the same roof and sharing facilities with an index case, for at least 2 weeks before index case’s TB diagnosis was made. An index case was any individual aged above 13 years, who was diagnosed with smear positive TB in the preceding 1 month and was still on the intensive phase of anti-TB therapy. We excluded those with pre-existing co-morbid chronic illnesses like cerebral palsy, congenital cardiac disease and diabetes mellitus.

### Baseline data collection and longitudinal follow-up

Purposive sampling of consecutive child household contacts was done. Enrollment continued until minimum sample was obtained. Administered structured questionnaires sought information on index case TB treatment, socio-demographics and TB knowledge. Knowledge sought covered causation, risk factors and prevention. A Likert Scale was used to categorize total appropriate scores into good, average or poor knowledge.

Contacts underwent baseline clinical evaluations by a qualified pediatrician, using the Kenya DLTLD pediatric TB clinical screening guidelines [15]. ‘Weight for age’ was used to asses chronic malnutrition, and those <60% were classified as severe, while those between 60 and 80% as moderate malnutrition. Weight faltering (failure to gain weight or weight loss over two consecutive visits) was identified from growth charts. BCG vaccination status was confirmed by presence of a scar on the left lateral forearm. TB suggestive symptoms and signs included fevers >38 °C (≥2 weeks), cough (≥2 weeks), reduced play and malaise, weight faltering, lethargy, fatigue, and respiratory signs. Contacts underwent a baseline intra-dermal tuberculin skin test (TST) on the right proximal lateral forearm, using two tuberculin units of tuberculin purified protein derivative RT 23 (Statens Serum Institute), and horizontal diameter measured after 72 h as per WHO guidelines [16]. Latent tuberculosis infection was diagnosed on the basis of a positive TST (induration ≥5 mm in diameter, in HIV-infected children and ≥10 mm in HIV-uninfected children), in the absence of evidence of active TB disease. Symptomatic contacts underwent chest radiography and sputum sampling. TB suggestive radiology included apical consolidations, mediastinal widening, pleural effusions or milliary pattern. Any other consolidations were regarded as non-specific changes. Sputum samples were obtained by direct expectoration, by induction (by nebulization with 3% saline for 15 min) or by gastric aspiration. Sputum microscopy for acid alcohol fast bacilli (AAFBs) was done [16]. Mycobacterial cultures were not done. Those with suspected extra-pulmonary TB underwent specific evaluations at the referral laboratory whenever indicated including, lymph node aspirates, pleural aspirate analysis and histopathology. Symptomatic contacts were put on oral antibiotics and clinical re-evaluation done after 2 weeks. The final TB scoring was done using the pediatric TB score chart (Additional file 1). Those with total score > 7 were considered to have ‘active TB disease’. HIV status of contacts was confirmed by a dried blood spot sample for PCR for HIV DNA. All contacts underwent baseline liver enzymes assays.

Contacts were put on isoniazid tablets daily, at a dose of 10 mg/kg, which was broken to the nearest quarter or half, for 6 months. Thereafter, monthly follow-up visits were done for another 6 months, during which contacts were re-evaluated for evidence of new ‘active TB disease’ as the primary outcome. Those symptomatic had a repeat TST done, at least 3 months from the earlier test, and chest radiography. Secondary outcomes included compliance and safety screening. Compliance was assessed by pill counts returned and caregiver/self reports. Compliance >90% was considered optimal. Safety screening involved caregiver and/or self reports and a repeat liver enzyme assay after 1 month on isoniazid. Clients were encouraged to report any operational challenges they experienced, and were supported by community health volunteers to optimize compliance. IPT failure was defined as occurrence of incident ‘active TB disease’ in a child on isoniazid, whether still on medication or in follow-up phase.

### Data management and analysis

A sample-size calculation based on Fisher et al., (1998) formula [17], required a minimum of 387 contacts, but an additional 39(10%) were enrolled to cover attrition. Oversight and safety monitoring was done by the Kenyatta University-Ethics Review Committee (KU-ERC). Data was coded, entered and analyzed using the statistical software- SPSS version 19.1. Data on children whose guardians declined further study participation, and those that did not continue follow-up to 1 year were censored at the date of the last visit and excluded from the analysis. Analyses followed the protocol completion approach.

Baseline contacts and index case characteristics were presented as frequencies, percentages, means and ratios. Comparisons were made of independent variables observed in those with and without IPT failure. Due to the small numbers of incident ‘active TB disease’ cases, variables were collapsed into 2 × 2 tables. Cross tabulations were done, and associations established by determiningodds ratios. Inferential statistics (*p*-value, confidence intervals) were used to establish factors associated with occurrence of IPT failure. Statistical significance level was fixed at *p* < 0.05. For advanced statistics, factors showing significant associations on bivariate analysis were entered into a multiple logistic regression model, to establish those with an independent relationship with IPT failure.

## Results

### Subjects’ enrollment and participants

**Fig. 1:**
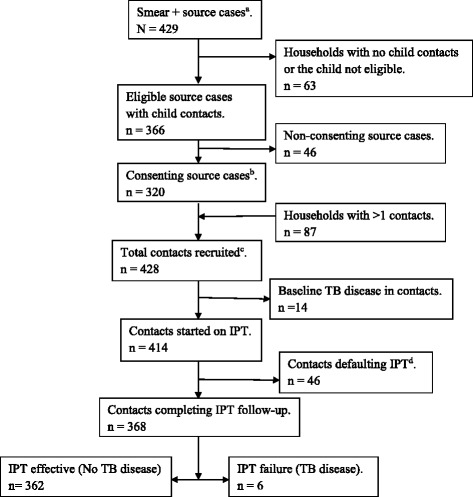
Subjects’ enrollment and participants. ^a^ Smear + = Sputum smear positive for acid alcohol fast bacilli, ^b^ Accepted to give the child isoniazid prophylaxis, ^c^ Contacts recruited are those that were eligible and consented to participate, ^d^ Contacts defaulting IPT are those that stopped the taking isoniazid, those that discontinued the 1 year follow-up program before endpoint, and those that transferred out of the study sites. TB tuberculosis, IPT Isoniazid preventive therapy

Out of 429 index cases diagnosed during the study period, 366 cases had household contacts aged below 5 years, hence eligible. IPT acceptance rate (consented) was 87.3% of the eligible source cases. Some (27.1%) households had >1 child per household, bringing to 428 contacts enrolled that underwent baseline screenings. Upon clinical scoring, 14(3.2%) contacts were diagnosed with ‘active TB disease’, hence excluded (Additional files 2 and 3). A total of 414 contacts were started on IPT. The flow chart of subjects’ enrollment and participants are presented in Fig. 1.

### Index case characteristics

All index cases that completed follow-up were on first line anti-TB treatment, though 26(7.1%) were re- treatment cases, having had a previous episode, that was fully treated and had been declared cured. Their age ranged between 13 and 62 years, with mean age of 26.7 years (SD = 8). The male to female ratio was 1:1. Majority, (85%) had secondary school level of education and below, 70.1% were unemployed, 76.4% were married and 74.7% were a biologic parent to the child. Most (72.2%) resided within informal settlements: 78.5% lived in single rooms and 74.1% lived in stone walled houses. The index case shared sleeping room with contacts in 80.9% cases, and 10.0% had >1 index case per household. The estimate time spent by index case at home per day was higher for females (mean = 16.7 h) compared to the males (mean = 12.5 h). Majority, (85.5%) used biomass fuels. We documented good knowledge level on TB causation in 70.9%, on risk factors for TB transmission to children in 32.3%, and on TB/HIV relationship in 55.1%. Seventy eight (21.2%) index cases tested HIV positive. Index case characteristics are presented in Table 1.

**Table 1:** Index case and household factors influencing IPT outcomes

Characteristics		TB disease	No TB disease	Fisher’s exact test (*P* value)	OR^a^ 95% CI
Age (years)	≤ 30	6	279	0.19	–
	>30	0	83		
Gender	Female	6	177	0.02^c^	–
	Male	0	185		
Residence	Informal settling	5	261	1.00	1.93
	Peri-urban	1	101		0.55 –16.77
Marital status	Married	5	276	1.00	1.55
	Single	1	86		0.18–13.52
Level of education	≤ Secondary	6	308	0.60	–
	Tertiary	0	54		
Occupation	Unemployed	2	256	0.068	4.83
	employed	4	106		0.87–26.77
Relationship to child	Parent	5	270	1.00	1.70
	Others^d^	1	92		0.19–14.77
Crowding index at night^e^	>5	4	74	0.02^c^	7.78
	≤4	2	288		1.39–43.31
Number of index cases per household	1	2	326	0.01^c^	0.54
	>1	4	35		0.01–0.30
Shared bedroom with contact	Always	5	293	1.00	0.85
	Sometimes	1	69		0.02–7.39
Separate Cooking room	Yes	1	79	1.00	1.40
	No	5	283		0.16 –12. 12
Duration of symptoms prior to TB diagnosis^b^	≤ 4 weeks	0	107	0.19	–
	>4 weeks	6	255		
HIV status	Positive	4	74	0.02^c^	7.78
	Negative	2	288		0.02–43. 32
Knowledge of TB causation	Bacteria	5	256	0.68	2.07
	Other	1	106		0.24–17.93
Knowledge of TB risk factors in children	Good/Average	3	116	0.64	2.07
	Poor	3	246		0.24 –17. 93
TB/HIV relationship knowledge^f^	Good/Average	3	200	0.04^c^	0.95
	Poor	3	304		0.11–8.32
TB myths and perceptions	Present	1	304	0.63	0.95
	None	5	58		0.11–8.32

^a^OR is presented in the top cell and CI in lower cell. ^b^ Time from onset of symptoms was calculated from the date the participant reported to start coughing until the date of treatment registered, ^c^ statistically significant factors. ^d^ Sibling, relative, or friend. ^e^ crowding index at night was obtained by number of all persons sleeping in the house divided by the number of rooms in the house, ^f^ Knowledge that HIV predisposes to tuberculosis. *CI* confidence interval, *OR* odds ratio, *TB* tuberculosis, *HIV* Human Immune Deficiency virus

### Characteristics of contacts completing IPT follow-up

Of 368 (88.8%) contacts that completed follow-up, 56.5% were aged <24 months, and their mean age was 21.4 months (SD = 5.6). The male to female ratio was 1:1. At least 41.8% were first born, 2.7% had low birth weight (<2500 g), and 63.3% were still breastfeeding. Majority, (79.6%) had accompanied parents to social places. At baseline screening, 34.2% had a TB suggestive symptom, 11.7% had malnutrition and/or growth faltering, 20.6% had a positive baseline TST, and 81.5% had a visible BCG scar. Twenty five (6.7%) tested HIV positive, but only 6(1.6%) were on anti-retroviral (ARV) therapy. Among reasons cited for this low rate ARV therapy among HIV positive children were adherence concerns, non-disclosure to other parent, denial, and fear of side effects. The baseline contacts’ characteristics are presented in Table 2 *.*

**Table 2:** Baseline characteristics of contacts completing IPT follow-up

Characteristic		(*N* = 368) N (%)^a^
Contact’s age (months)	≤ 24 months	208(56.5)
	> 24 months	168(43.5)
Gender	Male	189(51.1)
	Female	179(48.9)
Birth order	1st	154(41.8)
	2nd	126(34.2)
	≥ 3rd	88(23.9)
Birth weight (grams)	< 2500 g	13(2.7)
	2500–4000 g	316(85.9)
	> 4000 g	39(11.1)
Morbidity patterns	≥ 1 out-patient visit in 1 yr	50(13.6)
	≥ 1 hospital admission in 1 yr	41(11.1)
Breast feeding status	Exclusive breastfeeding	44(12.0)
	Currently breastfeeding	189(34.2)
	Not breastfeeding	135(36.6)
Weaning age (months)	Not yet weaned	44(12.0)
	< 4 months	128(34.7)
	4–6 months	196(53.3)
Nutrition status^b^	Weight faltering/underweight	43(11.7)
	Normal (> − 2 z-score)	325(89.9)
BCG vaccination	Scar present	300(91.5)
	Absent scar	68(18.5)
Visiting social places^c^	Yes	293(79.3)
	No	75(20.7)
TB suggestive symptoms ^d^	Yes	126(34.2)
	No	242(66.8)
Baseline TST reaction	Positive	76(20.6)
	Negative	292(79.4)
HIV DNA PCR	Positive	25(6.7)
	Negative/not done	343(93.3)

^a^Frequencies are presented in absolute number and percentages, ^b^ malnutrition was present in those with any weight faltering on their growth charts and those who had under-nutrition <80%, ^c^ Social places visited included churches, mosques, market places, or schools. ^d^ TB suggestive symptoms included those with cough ***≥***2 weeks, fever ***≥***2 weeks, weight loss, fatigue, reduced play, or any swellings. *BCG* Bacille Calmette-Guerin vaccine, *HIV* Human Immune Deficiency virus, *DNA* Deoxyribonucleic acid, *PCR* polymerase chain reaction, *TB* tuberculosis, *TST* tuberculin skin test

### Characteristics of contacts with IPT failure

Six (1.6%) contacts developed IPT failure. TB diagnosis was made during the 5th month on IPT in one case, while five were diagnosed during follow-up. Two cases were aged <24 months. Five cases had the mother as source, but in one case it was a sibling. They all shared sleeping rooms with the index case. Symptoms reported included weight loss (4), fevers (4), cough (2) and malaise (5). Positive clinical signs included malnutrition/growth faltering (3), persistent fevers >38 °C (5), respiratory signs (4) and lymphadenitis (4). Five had a visible BCG scar. A positive baseline TST was documented in four cases, and five tested positive upon repeat. Three were HIV positive, and all six had abnormal chest radiology.

### Operational challenges and drug related factors

Mothers were the main drug administrators (76.9%) and most, (42.9%) gave the drug in the evenings. Operational challenges were reported by 70.1% households. These included long treatment duration (65.2%), stock-outs (57.9%) and difficulties in administering tablets to children (44.3%). Side effects were reported in 22.2%, which included skin rash (15.2%), gastro-intestinal side-effects (nausea, anorexia, vomiting: 9.5%) and neurologic symptoms (irritability and/or weakness, paraesthesias, painful limbs, reduced play and altered sleeping patterns) in 5.4%. Six (1.6%) contacts had yellow discoloration of eyes or urine. The baseline mean serum glutamate pyruvate transaminase (sGPT) level was 46.1mmo/l, while mean serum glutamic-oxaloacetic transaminase (sGOT) level was 29.6 mmol/l. After 1 month on IPT, both mean sGPT and sGOT raised to 90.9mmo/l and 54.1 mmol/l respectively, a 2-fold increase. Severe hepatotoxicity (3-fold increases) was reported in only three contacts (0.08%), who were all on ARV therapy. Good compliance was observed in 89% (CI 80.2–96.2%) (Additional file 4).

### Factors associated with IPT failure

On univariable analysis, index case and household factors associated with IPT failure were, female gender (p = 0.01), crowding index >5 at night (p = 0.02), >1 source cases per household (p = 0.01), positive HIV status (p = 0.02) and knowledge of TB/HIV relationship (p = 0.04), (Table 1). Contact factors associated with IPT failure were malnutrition/weight faltering (p = 0.02), TB suggestive symptoms at enrollment (p = 0.02), positive HIV status (p = 0.01) and a positive baseline TST test (p = 0.02), (Table 3). None of the drug factors were associated with IPT failure. Independent variables that showed statistical significance were entered into the logistic regression model to determine those that had an independent relationship with IPT failure. Only malnutrition of contact remained significant (p = 0.05, CI 0.99-200.24), (Table 4). We re-analyzed our data excluding the HIV positive contacts, who may have responded differently to IPT, to determine factors correlating to IPT failure in non-infected children. However, none of the factors showed significant associations with IPT failure.

**Table 3:** Contact factors influencing IPT outcomes

Characteristics		TB disease	No TB disease	Fishers’ exact test (P value)	OR^a^ (95% CI)
Gender	Male	2	187	0.44	2.14
	Female	4	175		0.39 –11.81
Contact age (months)	≤ 24 months	2	204	0.70	1.55
	>24 months	4	158		0.28–8.57
Nutrition status^c^	Malnutrition/ Weight faltering	3	40	0.02^b^	0.124
	Normal	3	322		0.024–0.64
BGC scar	Positive	5	295	1.00	1.14
	Negative	1	67		0.13–9.88
Breastfeeding currently	Yes	3	230	0.67	1.74
	No	3	132		0.35–8.76
Appropriate weaning time	Yes	4	236	1.00	1.07
	No	2	126		0.19–5.91
TB suggestive symptoms at enrollment^d^	Yes	5	121	0.02^b^	0.10
	No	1	241		0.01–0.87
Birth weight	LBW (≥2500 g)	1	12	0.20	5.83
	Normal (>2500 g)	5	350		0.63–53.86
Recent morbidity in last 3 months	Yes	1	40	0.51	0.62
	No	5	322		0.07–5.45
Hospital admissions in last 1 year	Yes	5	49	0.59	0.78
	No	1	313		0.09–6.84
Social place^e^ attendance	Yes	5	288	1.00	0.78
	No	1	74		0.90–6.74
Baseline TST	Positive	4	72	0.02^b^	0.12
	Negative	2	290		0.02–0.69
HIV DNA PCR	Positive	3	22	0.01^b^	0.06
	Negative	3	340		0.01–0.34

^a^ OR is presented in the top cell and CI in lower cell. ^b^ Statistically significant factors, ^c^ malnutrition was present in those with any weight faltering on their growth charts and those who had under-nutrition <80%, ^d^ TB suggestive symptoms included those with cough ***≥***2 weeks, fever ***≥***2 weeks, weight loss, fatigue, reduced play, or any swellings. ^e^ Social places included churches, mosques, market places, or schools. *TB* tuberculosis, *BCG* Bacille Calmette-Guerin vaccine, *LBW* Low birth weight, *HIV* Human Immune Deficiency virus, *DNA* Deoxyribonucleic acid, *PCR* polymerase chain reaction, *TST* tuberculin skin test, *CI* confidence interval; *OR* odds ratio

**Table 4:** Variables in the equation on logistic regression

	B	S.E.	Wald	df	Sig.	Exp (B)	95% C.I. for EXP (B)	
							Lower	Upper
Index cases per household^a^							0.013	1.199
Crowding index >5 at night^b^	0.017	0.373	0.002	1	0.963	0.983	0.473	2.044
Index case HIV status	−1.299	1.517	0.734	1	0.392	0.273	0.014	5.330
Malnutrition/ weight faltering^c^	2.649	1.352	3.836	1	0.050^e^	14.137	0.998	200.237
Contact’s HIV DNA PCR test	2.504	1.705	2.157	1	0.142	12.225	0.433	345.312
Baseline TST reaction	2.275	1.312	3.006	1	0.083	9.731	0.743	127.444
Gender of index case	−18.892	2295.193	0.000	1	0.993	0.000	0.000	.
TB suggestive symptoms^d^ at enrollment	−14.923	4064.238	0.000	1	0.997	0.000	0.000	.
Side effects reported in child	−2.922	1.930	2.292	1	0.130	0.054	0.000	–
Constant	32.920	4667.541	0.000	1	0.994	1.981	–	–

^a^ Number of smear positive index cases in the household in the preceding 3 months, ^b^ crowding index at night was obtained by number of all persons sleeping in the house divided by the number of rooms in the house, ^c^ malnutrition was present in those with any weight faltering on their growth charts and those who had under-nutrition <80%, ^d^ TB suggestive symptoms included those with cough ≥2 weeks, fever ≥2 weeks, weight loss, fatigue, reduced play, or any swellings. ^e^ statistically significant factors. BCG Bacille Calmette-Guerin vaccine, HIV Human Immune Deficiency virus, DNA Deoxyribonucleic acid, PCR polymerase chain reaction, TB tuberculosis, TST tuberculin skin test

## Discussion

We sought to establish risk factors associated with IPT in children in HHC with smear positive TB, to help guide optimal preventive interventions. Malnutrition of contacts was the only independent factor significantly associated with IPT failure (p = 0.05). Forty three (11.7%) contacts had malnutrition/growth faltering. Malnutrition is associated with immune suppression, which leaves the child susceptible to mycobacterial invasion and disease progression [18]. However, limited studies have evaluated this risk. Moreover, TB infection itself creates an inflammatory state that further weakens the immune system [18]. Physical or emotional stresses are triggers to progression of infection to disease. Malnutrition is one of the stressors and increases risk of progression to disease by 2–5 times [19]. We did not find any published studies evaluating role of malnutrition on IPT strategy. However, our findings suggest that nutrition support of contacts may optimize IPT benefits in resource restricted settlements.

About 21.2% of index cases and 6.7% of contacts were HIV-infected. HIV positivity of contacts was not independently associated with IPT failure in our study. HIV exposed infants have been reported to have a 2-fold higher risk of TB exposure, than non-exposed infants, and HIV infection results in progression to relevant TB disease more frequently and more rapidly, with a greater likelihood of disseminated and extrapulmonary disease [6, 20]. However, the benefit of IPT in HIV exposed children is uncertain. Among South African children, IPT given for primary prophylaxis was found to have early survival benefit and reduced incidence of TB in children with HIV [21], with consequent recommendation for IPT use in HIV positive contacts [6]. However, in a recent meta-analysis, IPT was associated with sub-optimal benefit among HIV-infected children: a strong protective effect of isoniazid against TB among HIV-negative children (RR = 0.55, 95% CI 0.40, 0.75 *p* = 0.001), but no evidence of an effect among HIV-positive children (RR = 0.86, 95% CI 0.41-1.81 *p* = 0.187) [12]. HIV infected children are also more likely to be malnourished, which may be the indirect association with increased risk of failure. Early initiation of antiretroviral therapy and aggressive nutritional support could improve IPT benefits in HIV infected children. Unfortunately, most HIV infected children in our study had not been initiated on anti-retroviral therapy. When we re-analyzed our data excluding HIV infected contacts, none of the factors was significant. A possible explanation is that the small numbers of uninfected contacts with IPT failure could not produce a meaningful analysis.

In this cohort, a positive baseline TST test was associated with IPT failure on univariable analysis (*p* = 0.01). A positive baseline TST reflected prior TB exposures in these young contacts. Four contacts with IPT failure had a positive baseline TST, so the disease may have arisen as progression of latent TB infection. The other two TST negative cases were probably new infections arising from new transmission. Likewise, presence of TB suggestive symptoms at enrollment (*p* = 0.02) was univariably associated with IPT failure. Lack of a specific TB confirmatory diagnostic test remains a barrier to identifying infected children during contact investigations. This may have seen contacts with TB disease receive monotherapy with isoniazid with subsequent progression of isoniazid resistant mycobacteria leading to subsequent presentation with active TB disease. The more reliable Gene X-pert testing was incorporated in the Kenya National TB control program in 2015, but access remains limited. Besides, difficulties in obtaining sputum samples from children limits its use as a community based child TB screening tool [22].

Young children have immature immune systems, which predisposes them to disease progression. Despite continuous TB exposures in this cohort, young age (<24 months) was not independently associated with IPT failure (*p* = 0.70). Various studies have reported sub-optimal benefit among the very young [11, 12, 14]. In South Africa, there was no significant IPT benefit among very young HIV exposed infants on continuous IPT and antiretroviral therapy for primary prophylaxis [13]. One possible explanation for the discrepancy between findings is that children in this study already had TB exposure; hence IPT provided secondary prophylaxis. Presence of a BCG scar and ongoing breastfeeding also had a protective benefit. Both strategies should be promoted as part of primary TB prevention among HHC aged below 5 years, living in similar settings.

Five cases of IPT failure arose during the follow-up phase, at which time the index case had completed their anti-TB therapy. Similar findings were reported in the study by Martinson et al.*,* which compared four secondary prophylaxis regimens among persons with TB and HIV infection in high-burden settings. Rates of new TB in the continuous-isoniazid group markedly increased when therapy was discontinued. Exogenous re-infections may have occurred after IPT was stopped, but proving re-infection is difficult, since genotyping of initial and subsequent *M. tuberculosis* isolates was not done [23].

In TB endemic areas, duration and intensity of exposure might be a critical factor affecting IPT effectiveness. In our study, female gender of index case was univariably associated with IPT failure (*p* = 0.02). As expected, females spend more time with contacts than their male counterparts, hence increased exposure. Crowding index of >5 at night (p = 0.02) and presence of >1 source case per household (*p* = 0.01), which were both a reflection high degrees of exposures, were significantly associated with IPT failure. This corroborates findings that duration of infectiousness, infectivity of bacteria, intensity and proximity to infectious case may be used as measures of likelihood of infection [24, 25]. That these factors were not independently associated with risk of IPT failure could suggest that they are surrogate indicators of the level of nutritional support from parents, indicated by closeness of the mother as the source of food or inability to get adequate nutrition on account of the numbers that need to be fed. The positive univariable correlation of IPT failure with a positive HIV status of index case may be explained by the fact that HIV obscures TB presentation, resulting in delayed diagnosis, which prolongs contagious period [26].

Isoniazid was well tolerated, with 22.2% reporting minor side effects. Only three cases (0.08%) had significant hepatotoxicity. These contacts were also on ARV therapy, which may have exacerbated the liver insult. Safety of isoniazid was similarly documented in a meta-analysis of RCTs evaluating IPT in non-HIV infected persons in low burden countries, where hepatotoxicity was observed in 0.36% and 0.52% on 6 months and 12 months isoniazid course respectively. Higher toxicity occurred in high risk children and blacks [11, 27]. In another trial, isoniazid-related hepatitis occurred in 0.5%, but significant liver toxicity was more likely among malnourished or those severely unwell at diagnosis [28]. In the meta-analysis by Ayieko et al., only 0.8% of participants had adverse effects that necessitated discontinuation of isoniazid [12]. In this study, presence of side effects was not associated with IPT failure (*p* = 0.43). Possible explanation is that most side effects reported were minor, and hence did not affect compliance, which would have influenced IPT effectiveness.

Operational challenges within health system are often barriers to adherence. Though, reported by 70.1% of households, this was not associated with IPT failure. A South African study observed that IPT program providers in resource poor health care centers contribute to poor compliance as patients are more likely to be subjected to long waiting times, interrupted drug supplies and worse interpersonal experiences with care providers [29]. Poor adherence is an important barrier to effective chemoprophylaxis. Fairly good completion rates (88.8%) and compliance rates (89.0%) were observed in this study. Moreover, sub-optimal compliance did not predict IPT failure. Traditionally, drug adherence rates (based on taking >80% of prescribed doses) have been used to describe adherence to TB prophylaxis [30]. In various RCTs, compliance ranged from 50 to 75% through 1 year of preventive therapy [31–33]. However, both self report measures and clinic based pill counts tend to overestimate adherence, as death and lost-to-follow represent extreme non-adherence [34, 35]. In a South African study done in controlled settings, 78.6% children achieved a mean adherence >90% [36], but from studies in operational settings in Cape Town, only 15–27% achieved optimal adherence [31, 33]. Client education and support help optimize adherence and therefore IPT benefits realized. On the other hand, HIV co-infection often results in complex dosing schedules, toxicity, pill burden and high financial costs which reduce adherence [37, 38].

The baseline TB prevalence in HHC was 3.2%. Higher rates were reported in the Ugandan study at 10% [14]. A systematic review of child contact investigations in low and middle income reported a rate of 7% [39]. Intensive index case screening programs that had been implemented at our study sites may have contributed to the lower rates observed. By endpoint, incidence of new TB among contacts on IPT was 1.6%. A similar incidence was reported in the Ugandan study examining IPT benefit in children less than 15 years, at 1% [14]. However, our cohort looked at younger children below 5 years.

The risk of selection bias in this study arises from exclusion of transfers-out and those lost to follow-up from analysis for technical and logistical reasons. Community health volunteers were assigned to offer support whenever feasible, to minimize this attrition. Secondly, any index case still testing smear positive at end of 1 month of TB treatment was transferred-out to the referral center, and were henceforth excluded from contacts’ clinics. Our study could not therefore assess the contribution of isoniazid and/or multidrug resistant mycobacteria in index case to IPT failure. We could not also correlate index case TB treatment success versus failure and contact’s response, as all participating index cases were considered to have been cured after 6 months anti TB therapy.

Our analysis focused on IPT effectiveness as per protocol fulfillment. We observed 12.7% non-acceptance and 12.2% non-completion rates. Key contributors to this state were the changing dynamics within informal settlements. These included frequent migrations leading to transfers-out, lack of time to attend clinics as some index cases had to fend for their family livelihoods, and programmatic operational draw-backs within public health systems. The effectiveness of IPT by intention to treat was therefore much lower. This means that many child contacts who qualified for IPT, did not actually receive it. This presented missed opportunities to prevent child TB. Similar observations were reported in a study done in Ethiopia, which documented many such missed opportunities among child contacts in operational settings [40]. Secondly, 7.1% index cases in this cohort were re-treatment cases. They were screened for drug resistance by sputum microscopy and mycobacterial cultures at diagnosis and 1 month after initiation of TB therapy, and were still sensitive to first line anti-TB therapy. We however, could not asses the contribution of drug resistance to IPT failure. Another limitation is that TB diagnosis was made by clinical score charts. Generally, definitive TB diagnosis is very difficult in children [41], particularly in infants. This may have led to mis-diagnosis or over- diagnosis of TB in contacts. TB control programs should adopt stringent diagnostics to intensify identification of contacts unsuitable for IPT. We recommend larger studies to evaluate the contribution of mycobacterial drug resistance to IPT failure.

## Conclusion

There is high burden of TB infection and disease among under five household contacts with infectious TB cases in high burden settings. Contacts’ screening and IPT strategy reduces incidence of new active TB disease in exposed contacts. Malnutrition of contacts was associated with IPT failure. Benefits could be optimized through providing nutritional supports to child contacts at risk.

## Additional files

Additional file 1: Figure S1. Modified pediatric TB clinical score chart. (DOCX 13 kb)

Additional file 2: Table S1. Contact factors associated with active TB disease at baseline. (DOCX 14 kb)

Additional file 3: Table S2. Index case factors associated with active TB disease at baseline. (DOCX 16 kb)

Additional file 4: Table S3. Factors influencing compliance to isoniazid therapy. (DOCX 16 kb)

## Abbreviations

BCG: Bacille Calmette-Guerin (vaccine)
HHC: Household contacts
HIV: Human Immunodeficiency Virus
IPT: Isoniazid prophylaxis therapy
RCT: Randomized controlled trial
TB: Tuberculosis
TST: Tuberculin skin test
WHO: World Health Organization
