# Supplementary material for: Correlates of isoniazid preventive therapy failure in child household contacts with infectious tuberculosis in high burden settings in Nairobi, Kenya – a cohort study
Source: BMC Infect Dis. 2017 Sep 16;17:623. doi: 10.1186/s12879-017-2719-8 (PMC5602922; doi:10.1186/s12879-017-2719-8)
Supplement: Supplementary file 1 — Modified pediatric TB clinical score chart. (DOCX 13 kb) [file 12879_2017_2719_MOESM1_ESM.docx]

## Additional file 1

## *Figure S1. Modified pediatric TB clinical score chart.*

TB smear positive index case (3)

Age < 2years (1) BCG scar present (- 1)

**Suggestive history** Cough ≥ 2 weeks (1) Weight loss (>60-80%)/ weight faltering (1) Severe malnutrition (<60% ) (3) Unexplained fever ≥ 2weeks or night sweats (1) Fatigue, reduced playfulness, less active (1)

**Suggestive examination findings** Painless lymphadenopathy, with or without sinus (3) Respiratory symptoms despite adequate antibiotics (2) Spine deformity or firm non- traumatic joint swellings (3) Unexplained abdominal swelling or ascites (3) Change in temperament, fits or coma (3)

**Suggestive investigations findings** Suggestive chest X-ray (3) Non-specific chest X-ray (2) TST positive (3) Sputum positive (3) Suggestive histology/cytology (3)

## (Adapted from Kenya DLTLD pediatric TB score chart and the Kenneth Jonne’s criteria)
