# Supplementary material for: Correlates of isoniazid preventive therapy failure in child household contacts with infectious tuberculosis in high burden settings in Nairobi, Kenya – a cohort study
Source: BMC Infect Dis. 2017 Sep 16;17:623. doi: 10.1186/s12879-017-2719-8 (PMC5602922; doi:10.1186/s12879-017-2719-8)
Supplement: Supplementary file 2 — Contact factors associated with active TB disease at baseline. (DOCX 14 kb) [file 12879_2017_2719_MOESM2_ESM.docx]

## Additional file 1

***Table S1. Contact factors associated with active TB disease at baseline.***

| **Characteristics** | | **TB disease**  **(n= 14)** | **No TB disease**  **(n= 414)** | **Fisher’s exact test (P- value)** | **Odds ratio^a^**  **(95% CI)** |
| --- | --- | --- | --- | --- | --- |
| **Contact age** | < 24months | 10 | 223 | 0.243 | **1.792** |
|  | >24months | 4 | 167 |  | 0.553- 5.811 |
| **Gender of contact** | Female | 5 | 196 | 0.419 | **0.578** |
|  | Male | 9 | 204 |  | 0.190 – 1.756 |
| **Nutrition^b^ status of contacts** | Normal | 3 | 349 | 0.000 | **0.40** |
|  | Malnutrition/ Weight faltering | 11 | 51 |  | 0.011- 0.148 |
| **BGC scar** | Positive | 4 | 326 | 0.000 | **0.091** |
|  | Negative | 10 | 74 |  | 0.028 – 0.297 |
| **Birth weight** | LBW | 6 | 16 | 0.000 | **18.000** |
|  | Normal | 8 | 384 |  | 5.58 - 58 |
| **Currently breast feeding** | No | 5 | 143 | 0.619 | **0.998** |
|  | Yes | 9 | 257 |  | 0.328- 3.036 |
| **Weaning time (months)** | Appropriate (≥ 6 ) | 4 | 254 | 0.061 | **0.230** |
|  | Inappropriate(<6) | 10 | 146 |  | 0.110 – 0.746 |
| **Baseline TST test** | Positive | 9 | 86 | 0.001 | **6.86** |
|  | Negative | 5 | 328 |  | 1.047- 8.446 |
| **HIV DNA PCR test** | Positive | 5 | 22 | 0.000 | **2.141** |
|  | Negative | 9 | 378 |  | 1.044 – 3.447 |
| **Morbidity in last 3 months** | Yes | 14 | 343 | 0.000 | - |
|  | No | 0 | 57 |  |  |
| **Social places^c^ attendance** | Yes | 14 | 76 | 0.056 | - |
|  | No | 0 | 324 |  |  |

^a^ = OR is presented in the top cell and CI in lower cell,  ^b^ = malnutrition was present in those with any weight faltering on their growth charts and those who had under-nutrition <80%, ^c^ = social places visited included churches, mosques, market places, or schools. BCG Bacille Calmette-Guerin vaccine, CI confidence interval HIV Human Immune Deficiency virus, DNA Deoxyribonucleic acid, PCR polymerase chain reaction, TB tuberculosis , TST tuberculin skin test.
