# Supplementary material for: Correlates of isoniazid preventive therapy failure in child household contacts with infectious tuberculosis in high burden settings in Nairobi, Kenya – a cohort study
Source: BMC Infect Dis. 2017 Sep 16;17:623. doi: 10.1186/s12879-017-2719-8 (PMC5602922; doi:10.1186/s12879-017-2719-8)
Supplement: Supplementary file 3 — Index case factors associated with active TB disease at baseline. (DOCX 16 kb) [file 12879_2017_2719_MOESM3_ESM.docx]

## Additional file 1

***Table S2. Index case factors associated with active TB disease at baseline***

| **Characteristics** |  | **TB disease**  **(n= 14)** | **No TB disease**  **(n= 414)** | **Fisher’s exact**  **(p-value)** | **Odds ratio^a^** |
| --- | --- | --- | --- | --- | --- |
|  |  |  |  |  | **95% CI** |
| Age | ≤ 30 years | 11 | 308 | 0.594 | **1.095** |
|  | >30 years | 3 | 92 |  | 0.299– 4.000 |
| Gender | Female | 5 | 199 | 0.224 | **0.561** |
|  | Male | 9 | 201 |  | 0.185- 1.704 |
| Marital status | Married | 13 | 304 | 0.122 | **4.105** |
|  | Single | 1 | 96 |  | 0.530- 31.789 |
| Relationship with child | Mother | 11 | 299 | 1.000 | **1.239** |
|  | Other^d^ | 3 | 109 |  | 0.339 – 4.528 |
| Level of education | ≤ Secondary | 12 | 304 | 0.122 | **4.105** |
|  | > Secondary | 2 | 96 |  | 0.530- 31.789 |
| High risk social habits^e^ | Present | 5 | 150 | 0.566 | **0.926** |
|  | Absent | 9 | 250 |  | 0.305- 2.815 |
| Crowding index at night^f^ | ≥ 5 | 0 | 82 | 0.043^c^ | - |
|  | <5 | 14 | 311 |  |  |
| Number of index cases in household | ≥2 | 4 | 44 | 0.067 | **0.310** |
|  | 1 | 10 | 355 |  | 0.093 -1.030 |
| Share bedroom with child | Sometimes | 2 | 78 | 0.471 | **0.688** |
|  | Always | 12 | 322 |  | 0.151-3.137 |
| Residence | Slum | 8 | 293 | 0.153 | **0.487** |
|  | Peri-urban | 6 | 107 |  | 0.165 – 1.436 |
| Cooking fuel | Smoky | 11 | 347 | 0.291 | **0.560** |
|  | Non- smoky | 3 | 53 |  | 0.151- 2.073 |
| Cough symptom in index case | Yes | 8 | 320 | 0.049^c^ | **0.333** |
|  | No | 6 | 80 |  | 0.112 - 30.988 |
| Symptoms duration prior to TB diagnosis^b^ | ≤ 4 weeks | 11 | 122 | 0.000^c^ | **8.355** |
|  | > 4 weeks | 3 | 278 |  | 2.290– 30.481 |
| HIV status | Positive | 6 | 83 | 0.057 | **2.864** |
|  | Negative | 8 | 317 |  | 0.967 - 8.483 |
| TB treatment challenges | Present | 7 | 329 | 0.070 | **0.216** |
|  | Absent | 7 | 71 |  | 0.073 - 0.635 |
| Knowledge of TB spread | Good | 10 | 367 | 0.028^c^ | **0.225** |
|  | Poor | 4 | 33 |  | 0.067– 0.756 |
| Knowledge of TB causation | Good | 10 | 289 | 0.626 | **1.009** |
|  | Poor | 4 | 115 |  | 0.310 – 3.282 |

^a^ = OR is presented in the top cell and CI in lower cell. ^b^ = Time from onset of symptoms was calculated from the date the participant reported to start coughing until the date of treatment registered, ^c^ = Statistically significant factors. ^d^ =Sibling, relative, or friend. ^e^ = High risk social habits included alcohol intake, smoking, or singing, ^f^ = crowding index at night was obtained by number of all persons sleeping in the house, divided by the number of rooms in the house, CI confidence interval; OR odds ratio, TB tuberculosis, HIV Human Immune Deficiency virus.
