# Supplementary material for: Correlates of isoniazid preventive therapy failure in child household contacts with infectious tuberculosis in high burden settings in Nairobi, Kenya – a cohort study
Source: BMC Infect Dis. 2017 Sep 16;17:623. doi: 10.1186/s12879-017-2719-8 (PMC5602922; doi:10.1186/s12879-017-2719-8)
Supplement: Supplementary file 4 — Factors influencing compliance to isoniazid therapy. (DOCX 16 kb) [file 12879_2017_2719_MOESM4_ESM.docx]

## Additional file 1

***Table S3. Factors influencing compliance to isoniazid therapy***

| **Characteristics** |  | **Optimal compliance**  **(≥ 90%)** | **Sub- optimal compliance**  **(<90%)** | **Fisher’s exact (p value)** | **Odds Ratio^a^** |
| --- | --- | --- | --- | --- | --- |
|  |  |  |  |  | **95%CI** |
| Age of contact | ≤ 24 months | 180 | 28 | 0.753 | **0.866** |
|  | >24 months | 141 | 19 |  | 0.465- 1.615 |
| Nutrition status^b^ of contacts | Malnutrition / Weight faltering | 34 | 9 | 0.093 | **1.999** |
|  | Normal | 287 | 38 |  | 0.890 – 4.489 |
| HIV DNA PCR in contact | Positive | 20 | 5 | 0.201 | **1.792** |
|  | Negative | 301 | 42 |  | 0.638 – 5.028 |
| Occurrence of side effects | Yes | 222 | 34 | 0.399 | **1.166** |
|  | No | 99 | 13 |  | 0.590- 2.306 |
| Birth order | First born | 142 | 13 | 0.039^c^ | **2.075** |
|  | >Second | 179 | 34 |  | 0.055 – 4.79 |
| Index case age | ≤ 30 years | 246 | 39 | 0.454 | **0.673** |
|  | > 30 years | 75 | 8 |  | 0.301- 1.503 |
| Relation with child | Parent | 236 | 39 | 0.209 | **0.570** |
|  | Other^d^ | 85 | 8 |  | 0.256 -1.268 |
| Level of education | ≤ Secondary | 275 | 39 | 0.059 | **1.226** |
|  | Tertiary | 46 | 8 |  | 0.539 – 2.791 |
| Occupation of index case | Employed | 93 | 17 | 0.200 | **0.720** |
|  | Un-employed | 228 | 30 |  | 0.379 – 1.368 |
| Residence | Slum | 236 | 30 | 0.114 | **1.573** |
|  | Peri- urban | 85 | 17 |  | 0.826- 2.998 |
| Primary drug administrator | Mother | 251 | 34 | 0.463 |  |
|  | other | 70 | 13 |  |  |
| Crowding index^e^ during the day | ≥ 5 | 67 | 11 | 0.704 | **0.863** |
|  | < 5 | 254 | 36 |  | 0.417 -1.786 |
| Knowledge of TB causation | Yes | 236 | 25 | 0.044^c^ | **2.443** |
|  | No | 85 | 22 |  | 1.309 – 4.561 |
| Knowledge of TB/HIV relationship^f^ | Yes | 184 | 19 | 0.022^c^ | **1.979** |
|  | No | 137 | 28 |  | 1.061- 3.691 |
| TB myths/ perceptions | None | 270 | 39 | 0.832 | **1.086** |
|  | Yes | 51 | 8 |  | 0.480- 2.459 |

^a^ = OR is presented in the top cell and CI in lower cell. ^b^ = malnutrition was present in those with any weight faltering on their growth charts and those who had under-nutrition <80%, ^c^= Statistically significant factors. ^d^ = Sibling, relative, or friend. ^e^ = crowding index was obtained by number of all persons in the house divided by the number of rooms in the house, ^f^= Knowledge that HIV predisposes to tuberculosis. CI confidence interval; OR odds ratio, TB tuberculosis, HIV Human Immune Deficiency virus. DNA Deoxyribonucleic acid, PCR polymerase chain reaction, TST tuberculin skin test.
